# Abnormal Uterine Bleeding in Adolescence: When Menarche Reveals other Surprises

**DOI:** 10.1055/s-0041-1736143

**Published:** 2021-11-16

**Authors:** Helena Gomes, Bruna Abreu, Liliana Barros, Carlos Veríssimo

**Affiliations:** 1 Departament of Gynecology/Obstetrics, Hospital Beatriz Ângelo, Loures, Lisboa, Portugal

**Keywords:** abnormal uterine bleeding, adolescence, Burkitt lymphoma, hemorragia uterina anômala, adolescência, linfoma de Burkitt

## Abstract

**Introduction** Abnormal uterine bleeding is more frequent in adolescence. Although, most commonly, it has a non-structural etiology, it may be due to any cause described.

**Clinical case** A 12-year-old adolescent, with no relevant personal history, menarche 1 month before, was observed in the emergency department for severe menstrual bleeding with progressive worsening, and hemodynamic repercussion in need of transfusion support. Physiological ovulatory dysfunction associated with possible previously unknown coagulopathy was considered to be the most likely diagnosis and medical treatment was initiated. Without response, the patient was submitted to sedated observation and uterine aspiration, which ultimately led to the diagnosis of a Burkitt Lymphoma.

**Discussion** Although structural causes, and particularly malignancy, whether gynecological or not, are a rare cause of abnormal uterine bleeding in this age group, they must be considered, thus enhancing the fastest and most appropriate treatment.

## Resumo

**Introdução** A hemorragia uterina anormal é mais frequente na adolescência. Apesar de maioritariamente de etiologia não estrutural, pode dever-se a qualquer causa descrita.

**Caso clínico** Adolescente de 12 anos, sem antecedentes pessoais relevantes, com menarca há 1 mês, observada no serviço de urgência por hemorragia menstrual grave com agravamento progressivo e repercussão hemodinâmica com necessidade de suporte transfusional. Foi colocada a hipótese de disfunção ovulatória fisiológica associada a eventual coagulopatia desconhecida previamente e foi instituído tratamento médico. Por ausência de resposta a tratamento médico, foi submetida a observação sob sedação e aspiração uterina que evidenciou tratar-se de um Linfoma de Burkitt.

**Discussão** Apesar de as causas estruturais, e particularmente as neoplasias, do foro ginecológico ou não, serem uma causa rara de hemorragia uterina anormal nesta faixa etária, elas devem ser levadas em consideração potenciando assim um tratamento mais célere e adequado.

## Introduction

Abnormal uterine bleeding (AUB) and heavy menstrual bleeding (HMA) constitute a few of the main gynecological problems. They are more frequent in adolescence and one of the main reasons for referral to gynecology consultation in this age group. 1 2 3 4

The etiology of AUB can be structural or not. Nonstructural causes are more frequent in adolescence, mainly ovulatory dysfunction, physiological or not, followed by coagulopathies. 2 3 4 5 6 However, any cause described can occur at this age and a multifactorial etiology is common. Therefore, it is imperative to not exclude any cause before a thorough work-up simply because of age, even malignancies, which are rarely present. 5 6 7 8

## Clinical Case

We present the case of an adolescent, 12 years old, healthy, who had the menarche in the previous month with adequate menstrual flow for 7 days. She presented to the emergency room for menstruation with increased flow for 9 days (between 10 and 15 patches/day), and persistent vomiting. The patient had no other symptoms, especially those compatible with coagulopathy.

She was referred to gynecology due to progressive worsening of heavy menstrual bleeding and severe hemodynamic repercussion (severe anemia with Hb 7.3 g/dl, 117,000 platelets and a normal summary coagulation study). At the gynecological examination, an intact hymen and abundant vaginal bleeding with clots were observed. A rectal examination was performed, and it was suggestive of a mass on the posterior wall of the vagina, which on palpation worsened the blood loss through the vagina.

**Fig. 1:**
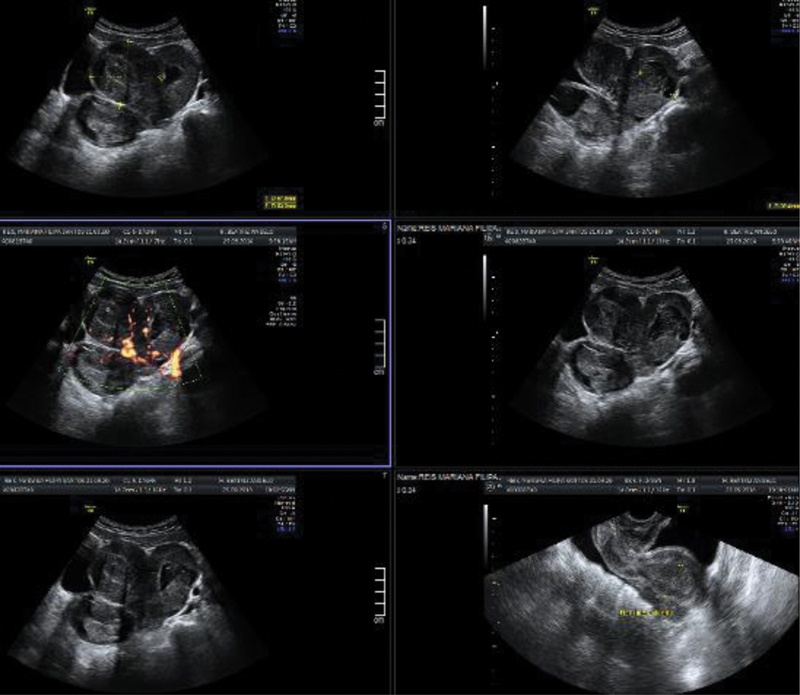
Abdominal and rectal ultrasound scan showing “bicorporal uterus, with a right hemicavidity with irregular endometrial thickening”.

Abdominal and rectal ultrasound was suggestive of a bicorporal uterus, with a noncommunicating left hemicavity with echogenic liquid content compatible with hematometra, and a right hemicavity with irregular endometrial thickening (24.8 mm of greater anteroposterior dimension); and an enlarged right ovary (57 × 41mm), dense stroma, and dispersed vascularization, without cystic formations ( Fig. 1 ).

**Fig. 2:**
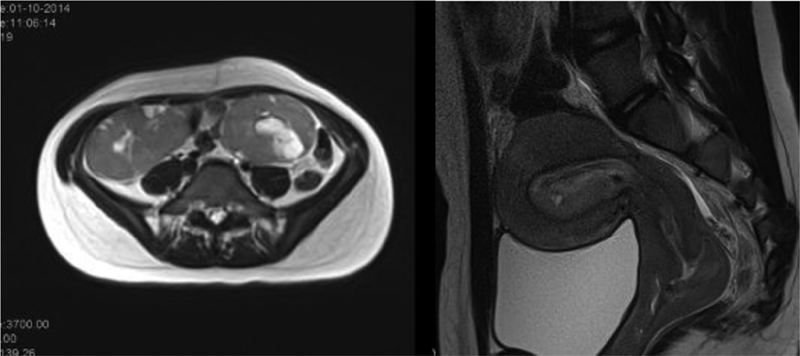
Computed tomography images with evidence of uterine and ovarian involvement due to previously diagnosed Burkitt lymphoma.

Abnormal uterine bleeding due to ovulatory dysfunction was admitted, eventually exacerbated by undiagnosed coagulopathy. The patient was started on antifibrinolytics (intravenous tranexamic acid), estroprogestative (high dose combined oral hormonal contraception) and transfusion support. Due to worsening of the hemorrhage, she underwent therapy with a gonadotropin agonist. On the 3 ^rd^ day of hospitalization, due to severe worsening with hemodynamic instability refractory to medical treatment, a gynecological examination was performed under sedation. A vagina with blood clots and a rough anterior wall, mainly in the upper third and hypertrophic single cervix, was observed. A partial aspiration of the right uterine hemicavity was performed, with significant hemorrhage reduction. The aspirated material was sent for analysis and the histological diagnosis was Burkitt lymphoma. Subsequently, complementary exams showed multiorgan impairment (hepatic, renal, breast, and ovarian). The patient was transferred to the Portuguese Institute of Oncology where the remaining diagnostic work-up also showed infiltration of the central nervous system. She was successfully treated with vincristine, cyclophosphamide, rituximab, methotrexate and arabinosine C, having been in remission for 5 years ( Fig. 2 ).

## Discussion

Abnormal uterine bleeding can be acute or chronic: acute requiring immediate intervention, whether episodic or in a chronic context; chronic if present in most of the preceding 6 months. 1 2 It affects between 10 and 20% of women and is more prevalent in adolescence. The evaluation of the menstrual cycle, as an additional vital sign, allows an early identification of an abnormal pubertal progression or, as exemplified in our case, the importance of the menstrual cycle as an initial manifestation of systemic disease. 3

Contrary to adult age, in adolescence the main causes of AUB are nonstructural, of which ovulatory dysfunction is the most frequent. When physiological, due to the immaturity of the hypothalamus-pituitary-ovary-axis, despite being a diagnosis of exclusion, it appears in more than ⅔ of the cases. When pathological, it occurs due to hyperandrogenism, hyperprolactinemia, hypothalamic or pituitary dysfunction or thyroid pathology. 1 2 3 4

Although rare in the general population (1%), coagulopathies are the second cause of AUB in adolescence: they are present in 20% of adolescents with AUB and in 30% of those in need of hospitalization. Heavy menstrual bleeding in menarche, even without a history of coagulopathy, is a frequent form of presentation, and in 50% of cases the first sign of a coagulation disorder. 5 6 7 8 9 The main associated coagulopathy is von Willebrand disease, and ∼ 13% of women with HMA have a variant of this disease. 2 4 Disorders of platelet function, coagulation factors, and thrombocytopenia are also prevalent in adolescence. 8 9

In this case, HMA in the first menstruation after menarche could lead to the presumptive diagnosis of coagulopathy. However, the absence of other symptoms of hemorrhagic dyscrasia and the analytical evaluation performed showed a low probability for this etiology.

As described, the first approach to a patient with acute AUB is to assess signs of hypovolemia and hemodynamic instability. Subsequently, the etiological investigation is performed according to the acronym PALM-COEIN (classification system approved by the Federation of Gynecology/Obstetrics): **P** olyps, **A** denomyosis, **L** eiomyomas, **M** alignancy and hyperplasia, **C** oagulopathy, **O** vulatory dysfunction, **E** ndometrium, **I** atrogenia and **N** ot classifiable. 2 4

Medical therapy should always be the first approach, being the only one necessary in 90% of cases of AUB in adolescents: hormonal, with high-dose combined oral contraception (or oral progestatives, if contraindicated for estrogens) or antifibrinolytic. The theoretical thrombotic risk in its association has not been proven and the medications should be combined if monotherapy fails. 4 10 11 Surgical therapy should be reserved for the failure of medical therapy or hemodynamic instability. 2 8

As recommended, in our case, in the face of AUB refractory to medical therapy with clinical instability, uterine aspiration was performed. This was a difficult decision, but one which allowed an early definitive histological diagnosis.

Intrauterine balloon tamponade (Foley catheter) could be an alternative, but with the disadvantages of not allowing histological diagnosis and having a proven use only in the postpartum period. 8 12

The use of gonadotropin agonists, even when refractory to medical therapy, is questionable. These play some role in severe chronic AUB but have limited use in acute AUB (due to flare up and response time). In this context, its use was due to the initial suspicion of a nonstructural cause and an attempt to avoid more invasive measures due to the age of the patient.

Burkitt lymphoma is a fast-growing tumor rarely diagnosed in adolescence. It can be classified as endemic, sporadic or associated with immunodeficiency. As a rule, it has a high 5-year survival rate (between 60 and 85%) but may have an adverse prognosis in the rare presence of genital involvement. Given its high response to chemotherapy, timely diagnosis and treatment is essential. 13 14 15

With the presentation of this case, we intend to alert to the approach of severe acute AUB in adolescence and to the possible less frequent structural etiologies in this age group. Although rare, the possibility of neoplasia must be considered in the diagnostic work up to enhance the appropriate treatment of our patients.
